# Reliability and validity of the total cerebral small vessel disease score: a systematic review and meta-analysis

**DOI:** 10.3389/fneur.2025.1593402

**Published:** 2025-06-27

**Authors:** Guilherme Diogo Silva, João Paulo Mota Telles, Carolina de Medeiros Rimkus, Germana Titoneli Vieira, Emily Figueiredo Vieira Neves Yuki, Raymundo Soares de Azevedo, Gisela Tinone, Leandro Tavares Lucato, Rosa Maria Pereira, Adriana Bastos Conforto

**Affiliations:** ^1^Department of Neurology, Hospital das Clínicas HCFMUSP, Faculdade de Medicina, Universidade de São Paulo, São Paulo, Brazil; ^2^Department of Radiology, Instituto de Radiologia, Hospital das Clínicas HCFMUSP, Faculdade de Medicina, Universidade de São Paulo, São Paulo, Brazil; ^3^Department of Rheumatology, Hospital das Clínicas HCFMUSP, Faculdade de Medicina, Universidade de São Paulo, São Paulo, Brazil; ^4^Department of Pathology, Faculdade de Medicina, Universidade de São Paulo, São Paulo, Brazil; ^5^Laboratory of Medical Investigation (LIM) 44, Hospital das Clínicas HCFMUSP, Faculdade de Medicina, Universidade de São Paulo, São Paulo, Brazil

**Keywords:** cerebral small vessel disease, score, reliability, validity, systematic review, meta-analysis, white matter hyper intensities, cerebral microbleed

## Abstract

**Introduction:**

Cerebral small vessel disease (CSVD) is a research priority to reduce the burden of stroke and dementia. The total cerebral small vessel disease (tSVD) score provides a global view of CSVD burden combining lacunes of presumed vascular origin, cerebral microbleeds, enlarged perivascular spaces, and white matter hyperintensities of presumed vascular origin. While its use in research is expanding, a systematic review of the tSVD score’s reliability and validity had not yet been undertaken. We reviewed the inter-rater and intra-rater reliability for the tSVD score and its features. We also examined the associations between the tSVD score and age, hypertension, stroke and cognitive impairment.

**Methods:**

We performed a systematic review of studies on Pubmed/MEDLINE, Embase, and Scopus databases from inception until June 21st, 2024. We included manuscripts that reported at least one of the following metrics for the tSVD or for its components: inter-rater reliability, intra-rater reliability, or associations with age, hypertension, stroke, and/or cognitive impairment. We provided summary Cohen’s kappa coefficients for inter and intra-rater reliability for each feature of the tSVD score. Subgroup analysis and meta-regression models were used to evaluate the impact of raters, MRI fields, age, and median tSVD score values in inter-rater reliability. We summarized studies reporting associations between the tSVD score, stroke and cognitive impairment.

**Results:**

The summary Cohen’s kappa values for inter-rater reliability ranged from 0.79 to 0.82 for each CSVD feature (13 studies, 8,177 participants). We found a high heterogeneity between studies (I^2^ = 94%), which may be explained by differences in rater, age, and median tSVD score. The summary Cohen’s kappa values for intra-rater reliability ranged from 0.78 to 0.84 (four studies, 250 cases were randomized from 3,654 participants). Heterogeneity was low. Seven studies (6,022 participants) reported associations between tSVD scores and either age or hypertension. Fifteen studies (6,941 participants) reported associations between tSVD scores and either stroke or cognitive impairment.

**Conclusion:**

The intra-rater reliability, inter-rater reliability, and construct validity of each feature of the tSVD support the use of this scale in CSVD research. However, inter-rater reliability might be influenced by rater characteristics, the median tSVD score, and participant age.

**Systematic review registration:**

https://www.crd.york.ac.uk/prospero/, identifier CRD42022372599.

## Introduction

Cerebral small vessel disease (CSVD), an intrinsic disorder of small perforating arterioles, causes one-fifth of ischemic strokes and contributes to 45% of dementia cases (1). The significant individual, social and economic burden of these conditions makes CSVD a research priority (2).

Classical measurements of CSVD rely on visual scales for white matter hyperintensities on neuroimaging (WMH) (3). However, CSVD affects both white and gray matter, with heterogenous ischemic and hemorrhagic brain damage (1). The Standards for Reporting Vascular Changes on Neuroimaging 2 (STRIVE-2) published in 2023 reinforced the need for summary measures of distinct CSVD features (4).

**Figure 1:**
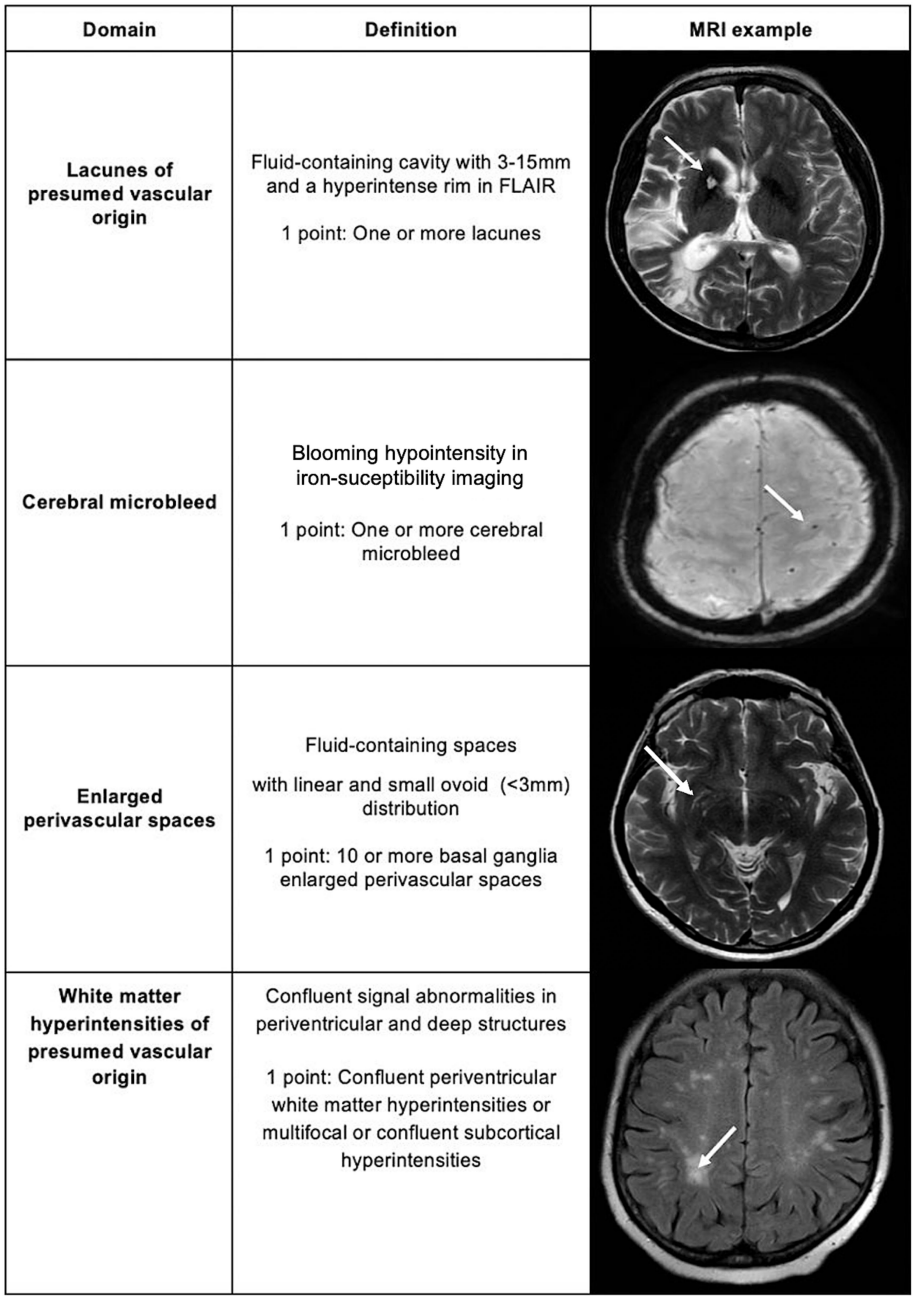
Total cerebral small vessel disease score.

The total cerebral small vessel disease score (tSVD score) provides a comprehensive view of CSVD (5). Raters evaluate brain magnetic resonance images (T1, T2, FLAIR, diffusion-weighted and iron-susceptibility imaging) and score 0 to 4 in an ordinal visual scale based on the presence of lacunes of presumed vascular origin (LAC), enlarged perivascular spaces (EPVS), cerebral microbleeds (CMB) and WMH of presumed vascular origin (Figure 1).

Despite its growing use in research, significant knowledge gaps remain regarding the tSVD score as a whole, as well as the performance of its individual features. First, no systematic review has yet evaluated its reliability and validity, a recommended step to ensure scientific rigor. Second, limited data exist on how the reliability of the tSVD score and its features may vary across different populations and among raters assessing SVD. Finally, further studies are needed to explore the clinical utility of the tSVD score and its features in predicting recurrent strokes across various stroke etiologies and its potential to predict dementia in diverse populations.

We conducted a systematic review to address the following research questions: Among adults with cerebral small vessel disease (Population), what is the reliability and validity (Outcomes) of the tSVD score and each of its features (Investigation), as assessed in observational studies (Study Design)? What factors influence the reliability of the tSVD score? What gaps exist in the current research on the reliability and validity of the tSVD score?

## Materials and methods

### Data sources

We performed a systematic review and meta-analysis compliant with the Preferred Reporting Items for Systematic Reviews and Meta-Analyses (PRISMA) (6). The study has been registered with the Prospective Register of Systematic Reviews (PROSPERO) under registration number CRD42022372599. The search strategy was “Total Cerebral Small Vessel Disease Score” OR “Total Small Vessel Disease Score” OR “tSVD score” OR “Total CSVD Score” in PubMed/MEDLINE, Embase and Scopus databases. Our final search was conducted on June 21, 2024, without any restrictions on publication year or language at this stage.

### Study selection

Included manuscripts reported at least one of following characteristics of the tSVD score or any of its features: inter-rater reliability, intra-rater reliability, or associations with age, hypertension, stroke or cognitive impairment. We excluded papers without accessible full text and non-English language manuscripts. We also excluded studies that used modified versions of the tSVD score in which any features were added or removed.

### Outcomes

Reliability, the agreement of two or more observations of the same entity (7), was evaluated by inter-rater and intra-rater agreement. Inter-rater agreement concerning the tSVD score or any of its features was measured by the comparison of scores from the same images by two independent raters. Intra-rater agreement was quantified on a single researcher’s repeat assessment of a sample of randomly selected brain scans. The effect sizes for the inter- and intra-rater reliability were measured with the Cohen’s kappa coefficient.

Validity was defined as the extent to which the results represent what they are supposed to measure (8). Validity of the tSVD score can be demonstrated by conceptual or operational strategies. It is reasonable to assume the tSVD score is valid based on a conceptual approach, given its features were derived from an expert consensus for reporting neuroimaging of CSVD (STRIVE) (1, 4). We used an operational strategy to assess construct validity, a review of the association between the tSVD score and age, hypertension, stroke and cognitive impairment. Both first-ever and recurrent strokes were considered, regardless of their causative classification. Cognitive impairment was included based on either a reported diagnosis of cognitive impairment or lower scores on screening tools such as the Montreal Cognitive Assessment (MOCA) scale. The effect size for the association between tSVD score, stroke and cognitive impairment was considered if the relative risk was higher than 2 or lower than 0.5 (9).

Two researchers independently reviewed titles and abstracts for eligibility using the Rayyan web-tool (10). In case of disagreement, consensus on which articles to screen full text was reached by discussion. Next, two researchers independently screened full-text articles for inclusion and, again, conflict was solved by consensus.

### Data analysis

Two reviewers independently extracted author, date and country of publication, age, sex, risk factors, complications, rater (neuroradiologist, radiologist, neurologist, neurosurgeon, other), inter-rater, and intra-rater agreement. Extracted data were compared, with any discrepancies being resolved through discussion.

We assessed the risk of bias with the Quality Appraisal Tool for Studies of Diagnostic Reliability (QAREL) (7). Two authors independently applied the tool to each study included for assessment of inter-rater and intra-rater reliability. A low risk of bias was considered if: (1) the samples included patients with CSVD; (2) the raters were neurologists, neuroradiologists, neurosurgeons, or radiologists; (3) the raters were blinded to the findings of other raters and to clinical information; (4) the raters assessed the same neuroimaging study; and (5) the tSVD score was applied according to the original description for each feature and summary score.

Continuous variables were summarized as median and interquartile ranges, whereas qualitative variables were described in percentages and total counts. When only a range of inter or intra-rater agreement was reported, instead of a point value, we included the mean value for statistical analysis. Cohen’s kappa coefficients between 0.61 and 0.80 were considered to indicate substantial reliability, while those between 0.81 and 1.00 were considered almost perfect (11). The standard error of the kappa statistics was calculated using a nomogram and sample size (12). We provided a table with a pooled estimate of the Cohen’s kappa coefficient for each feature of the tSVD score using a random effects model meta-analysis in R Studio 2022.07.2 with package meta.

The presence and extent of statistical heterogeneity in the pooled estimate of the Cohen’s kappa coefficient was calculated using I^2^. Subgroup analysis was performed to investigate the role of the type of rater (neuroradiologist versus other raters) and MRI field strength (3 Tesla versus 1.5 Tesla) as a potential source of heterogeneity. We conducted a meta-regression analysis to assess median age, sex (percentage of males), and the median tSVD score as potential contributors to heterogeneity.

We assessed the presence of publication bias using a funnel plot for intra-rater and inter-rater reliability. A sensitivity analysis was performed excluding the studies responsible for funnel plot asymmetry.

The certainty in the body of evidence for intra-rater and inter-rater reliabilities of tSVD score was assessed by the Grading of Recommendations, Assessment, Development, and Evaluations approach (9) (Summary of Findings table). Large observational studies were initially regarded as high-quality evidence for reliability studies. The quality of evidence was downgraded in the presence of: (1) risk of bias, measured by the QAREL risk of bias tool (7); (2) indirectness, considered if studies were conducted only in a particular setting (e.g., only primary care); (3) imprecision, defined by wide confidence intervals; (4) inconsistency, evaluated by high unexplained heterogeneity (I^2^ > 50%); and (5) publication bias, based on funnel plot analysis.

## Results

**Figure 2:**
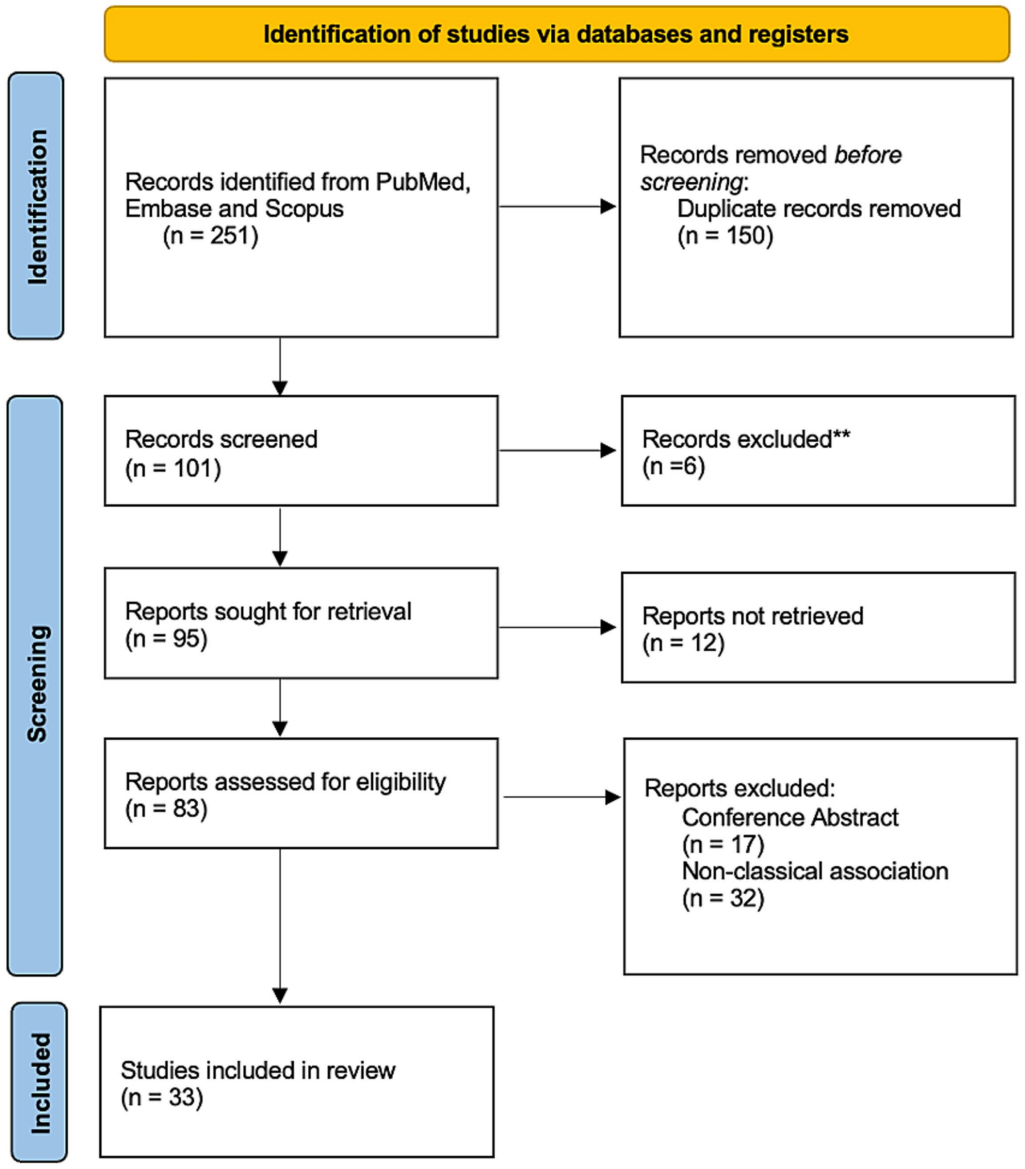
Flow diagram.

Figure 2 represents the flow diagram according to the PRISMA 2020 guidelines (6). We included 33 studies with 17,340 participants.

### Inter-rater reliability

**Figure 3:**
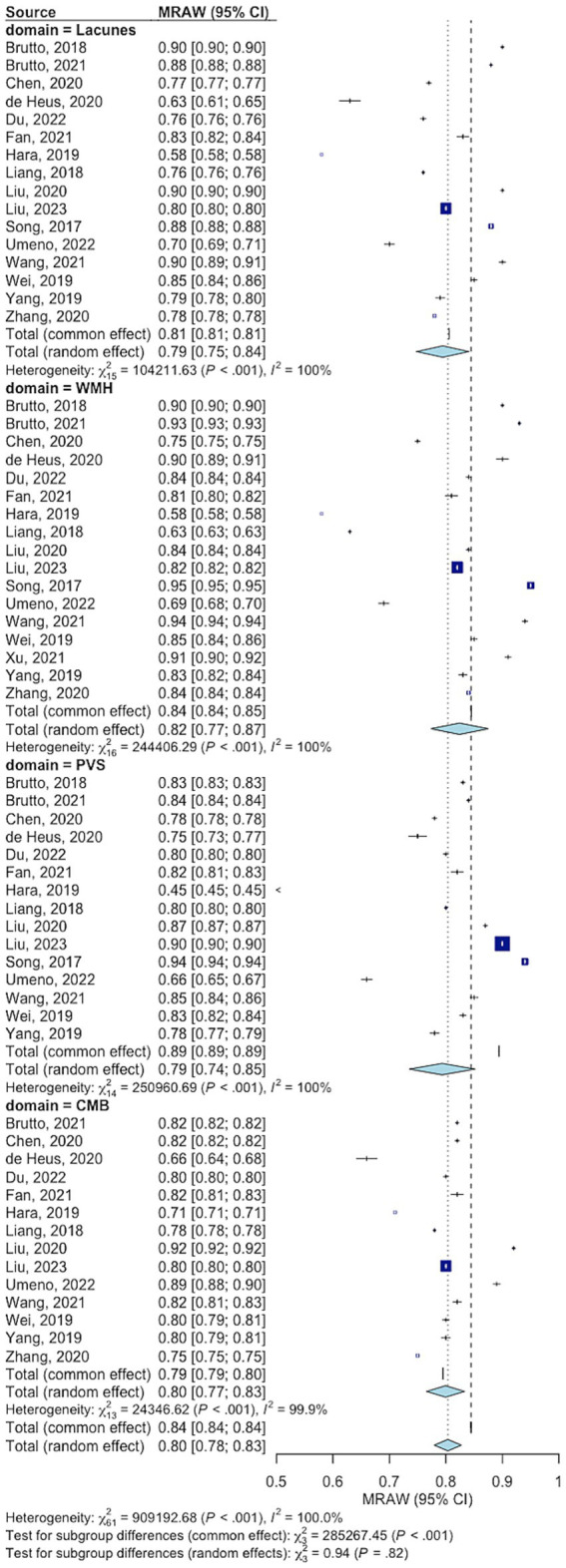
Summary Cohen’s kappa for inter-rater reliability of the total cerebral small vessel disease score by feature. WMH: White matter hyperintensity. CMB: Cortical microbleeds. EPVS, Enlarged perivascular spaces.

Inter-rater agreement was evaluated in 13 studies for a total of 8,177 participants (Supplementary Table S1) (13–25). All studies had low risk of bias based on the QAREL tool. Inter-rater reliability was reported for individual SVD markers but not for the total tSVD score across all studies. Pooled point estimates of Cohen’s kappa for LAC, WMH, EPVS, and CMB were classified as substantial or almost perfect (Figure 3). The most common cause of CSVD was sporadic CSVD in all included studies.

We found evidence of high heterogeneity between studies of inter-rater agreement, for each tSVD feature. We downgraded the quality of evidence based on inconsistency. A subgroup analysis of studies reporting the assessment of tSVD by neuroradiologists had a significantly lower heterogeneity (Supplementary Figure S1). The heterogeneity was high and comparable between studies using 1.5 and 3 Tesla MRI (Supplementary Figure S2). The meta-regression showed that older age and higher median tSVD score were linked to higher inter-rater agreement for cerebral microbleeds but did not affect agreements for the other features (Supplementary Table S2). Funnel plot showed a significant asymmetry (Supplementary Figure S3), a concern for publication bias. However, the Cohen’s kappa values for inter-rater agreement continued to be substantial or almost perfect after excluding studies responsible for funnel plot asymmetry in the sensitivity analysis (Supplementary Figure S4).

### Intra-rater reliability

**Figure 4:**
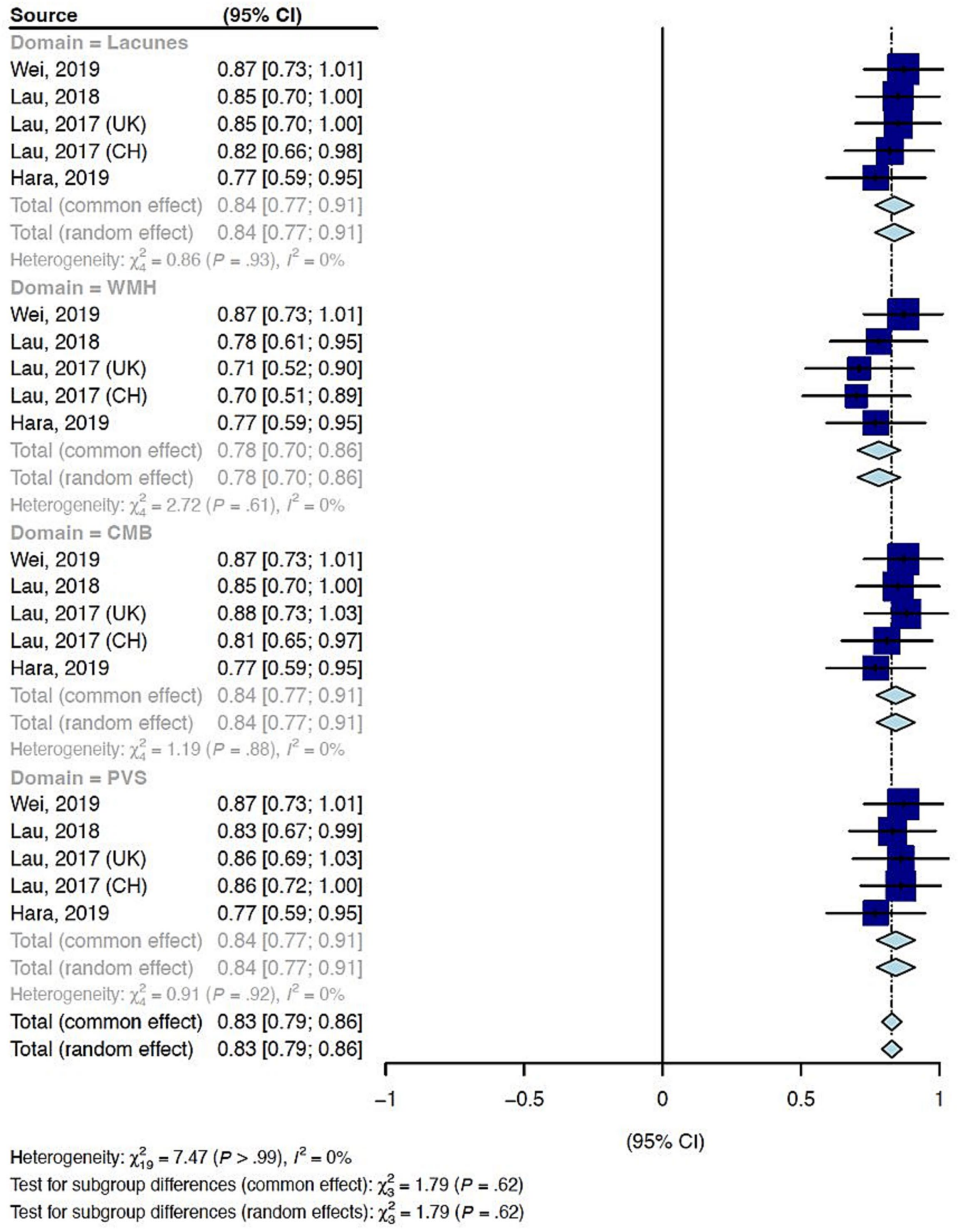
Summary Cohen’s kappa for intra-rater reliability of the total cerebral small vessel disease score by feature. WMH: White matter hyperintensity. CMB: Cortical microbleeds. EPVS, perivascular spaces.

Intra-rater agreement was assessed in four studies using 250 random cases for test and re-test methodology for a total of 3,654 participants (Supplementary Table S3) (17, 23, 26, 27). The risk of bias for intra-rater reliability studies was low. Intra-rater reliability was reported for individual SVD markers but not for the total tSVD score across all studies. The Cohen’s kappa coefficient indicated substantial reliability for WMH (0.78, 95% CI 0.70; 0.86), and almost perfect reliabilities for the other features (Figure 4).

Statistical heterogeneity measured by I^2^ was zero and funnel plots were symmetrical for all features of the tSVD score (Supplementary Figure S5). The most common cause of CSVD was sporadic CSVD in all included studies. Table 1 summarizes the summary measures and the certainty of evidence for inter-rater and intra-rater reliability of the tSVD score.

**Table 1:** Summary of reliability findings for each feature of the total cerebral small vessel disease score.

Number of participants (Studies)	Summary Cohen’s kappa for Lacunes (95% CI)	Summary Cohen’s kappa for WMH (95% CI)	Summary Cohen’s kappa for CMB (95% CI)	Summary Cohen’s kappa for EPVS (95% CI)	Certainty of the evidence (GRADE)	Comments
Intra-rater reliability						
Random 250 out 3,654 participants (Four studies)	0.84 (0.77; 0.91)	0.78 (0.70; 0.86)	0.84 (0.77; 0.91)	0.84 (0.77; 0.91)	⊕ ⊕ ⊕ ⊕ High	**–**
Inter-rater reliability						
10,795 participants (19 studies)	0.79 (0.73; 0.84)	0.82 (0.75; 0.88)	0.80 (0.77; 0.84)	0.79 (0.72; 0.86)	⊕ ⊕ ⊕◯ Moderate Due to inconsistency	Heterogeneity possibly caused by the different specialties of the raters

WMH, White matter hyperintensities; CMB, Cerebral microbleeds; EPVS, Enlarged perivascular spaces; GRADE, Grading of Recommendations, Assessment, Development, and Evaluations.

### Construct validity

The evaluation of the association between the tSVD score with age and hypertension was performed in seven studies involving a total of 6,022 participants (Supplementary Table S4) (14, 17, 19, 28–32). The associations between the tSVD score with stroke or cognitive impairment were reported in 15 studies involving a total of 7,996 participants (Supplementary Table S5) (15, 21, 22, 27, 29, 30, 33–42). A large effect size was observed in the association of tSVD score and hypertension, cardio-cerebrovascular events, and lacunar stroke (Supplementary Tables S4, S5).

## Discussion

The features of the tSVD score are reliable tool in the CSVD assessment. Cohen’s kappa’s values for inter and intra-rater agreements were substantial or almost perfect for WMH, LAC, CMB, and EPVS. The type of rater, age, and median score moderated the inter-rater reliability of the tSVD score. Age, hypertension, stroke, and cognitive impairment were linked to higher tSVD scores.

The use of highly reliable scores decreases the risk of type 2 errors (43). We classified the estimate Cohen’s kappa values as substantial or almost perfect for all of the features of the tSVD score. However, the reliability of a score depends on its raters. Notably, the raters in the studies reviewed were trained neuroradiologists, neurologists, neurosurgeons or radiologists. The statistical heterogeneity was lower in studies where tSVD scores were assessed exclusively by neuroradiologists. This finding highlights the influence of raters on tSVD reproducibility and underscores the potential need for standardized training on its application. All tSVD features were highly reliable, but the absence of studies reporting the intra- and inter-rater reliability of the total SVD score and the lack of individual-patient data limited the calculation of a summary reliability measure for the total SVD score. Future studies assessing the reliability of the tSVD score should report not only the reliability of each individual features but also the reliability of the overall tSVD score.

Our review primarily included studies from Asia, the United States, and Europe, with only a few from Latin America and none from Africa. It is crucial to validate the tSVD score across diverse populations, as ethnic differences in CSVD burden have been observed. Compared to Caucasians, Asians may have a higher prevalence of WMH (44), while MRIs from African American and Latin American subjects (45, 46) show a greater burden of CMB. Older age was associated with higher inter-rater reliability of the CMB in this study, but not the inter-rater reliability of other features of the tSVD score. Future research should further investigate how demographic characteristics influence the reliability of the tSVD score.

Non-arteriolosclerosis causes of CSVD are under-represented in the field, limiting the generalizability of the data for all causes of CSVD (47, 48). Other features may be important for non-arteriosclerosis causes of CSVD. Superficial siderosis, for instance, is relevant to evaluate the burden of cerebral amyloid angiopathy but is not included in the tSVD score (49). The tSVD score does not consider differences in the distribution of CSVD lesions among causes of CSVD. For example, in cerebral autosomal dominant arteriopathy with subcortical infarcts and leukoencephalopathy (CADASIL), WMH affect the extreme capsule and anterior temporal poles, uncommon in other causes of CSVD (50). All studies in the inter-rater and intra-rater analyses focused on populations with arteriolosclerosis as the primary cause of CSVD, restricting reliability and validity assessment to sporadic SVD.

The reliabilities of all tSVD features were substantial or almost perfect, while reliabilities of the periventricular and deep Fazekas scale were substantial (51). The tSVD score addresses the limitations of current methods for assessing CSVD burden on brain MRI. While the Fazekas scale addresses only WMH (51), the tSVD score integrates multiple CSVD markers – LAC, CMB, and EPVS.

The tSVD score provides a simple and practical measure of CSVD burden on brain MRI, in contrast with automatic segmentation tools that may offer greater precision but necessitates additional imaging acquisitions and processing (52). These requirements limit the widespread use of these techniques, particularly in retrospective studies that use routine clinical MRI data.

The tSVD score exhibits an evolving nature, leading to adaptations such as a simplified version that excludes EPVS, which are associated with dementia (53). Another adaptation incorporates centrum semiovale EPVS and cortical superficial siderosis, both of which correlate with pathological evidence of amyloid angiopathy (49). Other modifications include raising the EPVS threshold and refining the scoring for microbleeds and WMH burden based on their count and severity (54). This study focused on the original tSVD score, and future research should examine how its ongoing adaptations may affect the tool’s reliability and validity.

This meta-analysis relied solely on aggregated data; therefore, individual patient data were not available to assess other neuroimaging patterns of CSVD. Future studies in the field of CSVD should evaluate the tSVD score in subjects with non-atherosclerotic causes of CSVD, as well as its responsiveness to change over time.

The present study was limited to the two most used properties of a neuroimaging score: validity and reliability. Another relevant characteristic - responsiveness to change (55) - was not evaluated. Responsiveness to change is a concern for the tSVD score because LAC and CMB are scored according to their presence rather than their number. Hence, the tSVD score may carry a potential risk of a ceiling effect, as an increase in the number of LAC or CMB might not lead to a corresponding increase in the score. Moreover, the results of this systematic review suggest that higher median tSVD scores may be linked to higher inter-rater agreement for CMB. The tSVD score might be limited in at the extremes of the CSVD burden.

No longitudinal studies evaluating successive tSVD scores over time and its association with clinical symptoms were identified. Future longitudinal studies, particularly those leveraging big data and open science, should assess changes in the tSVD score over time and evaluate the impact of its increase on stroke and dementia risk. Additionally, adaptations of the tSVD score could include adjustments for the total number of lacunes and microbleeds, rather than just their presence, as a strategy to mitigate the risk of a ceiling effect.

## Conclusion

The tSVD score features are reliable and valid measures of CSVD, supporting its application in research. However, the inter-rater reliability of these features may be influenced by factors such as rater type, median tSVD score, and participant age. Future research should address several knowledge gaps, including the overall reliability of the tSVD score rather than just its individual features; the reliability and potential need for additional features in cases of CSVD unrelated to age and hypertension; and the optimal approach for longitudinal application of the tSVD score, particularly in light of potential ceiling effects.

## Data Availability

The original contributions presented in the study are included in the article/Supplementary material, further inquiries can be directed to the corresponding author.
