# Supplementary material for: Reliability and validity of the total cerebral small vessel disease score: a systematic review and meta-analysis
Source: Front Neurol. 2025 Jun 27;16:1593402. doi: 10.3389/fneur.2025.1593402 (PMC12247598; doi:10.3389/fneur.2025.1593402)
Supplement: Supplementary file 1 [file Supplementary_file_1.docx]

**Supplementary Material**

Title: Reliability and validity of the total cerebral small vessel score in vascular neurology: a systematic review and meta-analysis

Summary

[Figure S1. Subgroup analysis of inter-rater reliability for each feature of the total small vessel disease score by the specialty of the rater (neuroradiologist x others) 3](#_Toc197633531)

[Figure S2. Inter-rater agreement of the features of the total cerebral small vessel disease score in studies using 1.5T and 3T magnetic resonance imaging scanners 4](#_Toc197633532)

[Figure S3. Funnel plot for inter-rater reliability for each feature of the total cerebral small vessel disease score. A: Lacunes of presumed vascular origin B: White matter hyperintensities of presumed vascular origin. C: Cerebral microbleeds. D: Enlarged perivascular spaces. 5](#_Toc197633533)

[Figure S4. Sensitivity analysis excluding studies about inter-rater reliability that contributed to significant asymmetry in the funnel plot (Hara, 2019 and Liang, 2018) 6](#_Toc197633534)

[Figure S5. Funnel plot of intra-rater reliability for each feature of the total cerebral small vessel disease score. A: Lacunes of presumed vascular origin B: White matter hyperintensities of presumed vascular origin. C: Cerebral microbleeds. D: Enlarged perivascular spaces. 7](#_Toc197633535)

[Table S1. Studies reporting inter-rtater reliability of each domain of the total cerebral small vessel disease score 8](#_Toc197633536)

[Table S2. Meta-regression for age, sex, and median score to identify predictors of heterogeneity of the reliability of the total small vessel disease score 11](#_Toc197633537)

[Table S3. Studies reporting intra-rater reliability of each domain of the total cerebral small vessel disease score 12](#_Toc197633538)

[Table S4. Characteristics of studies reporting associations between the total cerebral small vessel disease score, age and hypertension. 13](#_Toc197633539)

[Table S5. Characteristics of studies reporting associations between total cerebral small vessel disease score, stroke, cognitive impairment, and cognitive tests 16](#_Toc197633540)

# **Figure S1.** Subgroup analysis of inter-rater reliability for each feature of the total small vessel disease score by the specialty of the rater (neuroradiologist x others)

**
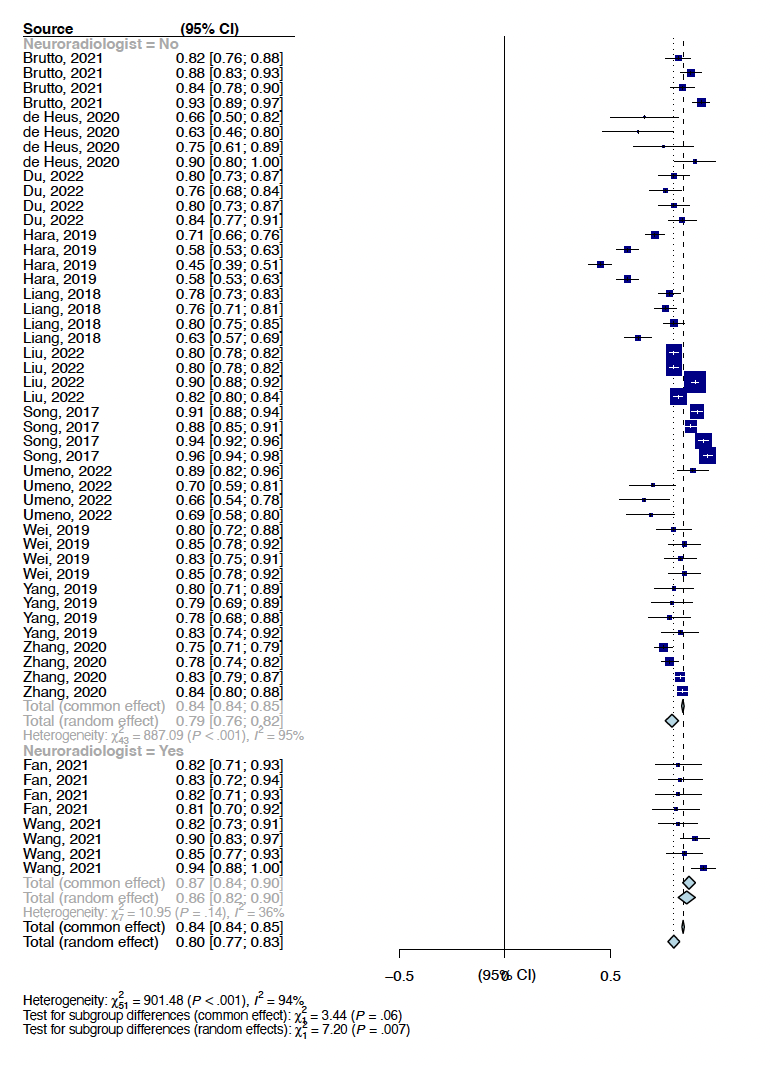
**

# **Figure S2.** Inter-rater agreement of the features of the total cerebral small vessel disease score in studies using 1.5T and 3T magnetic resonance imaging scanners


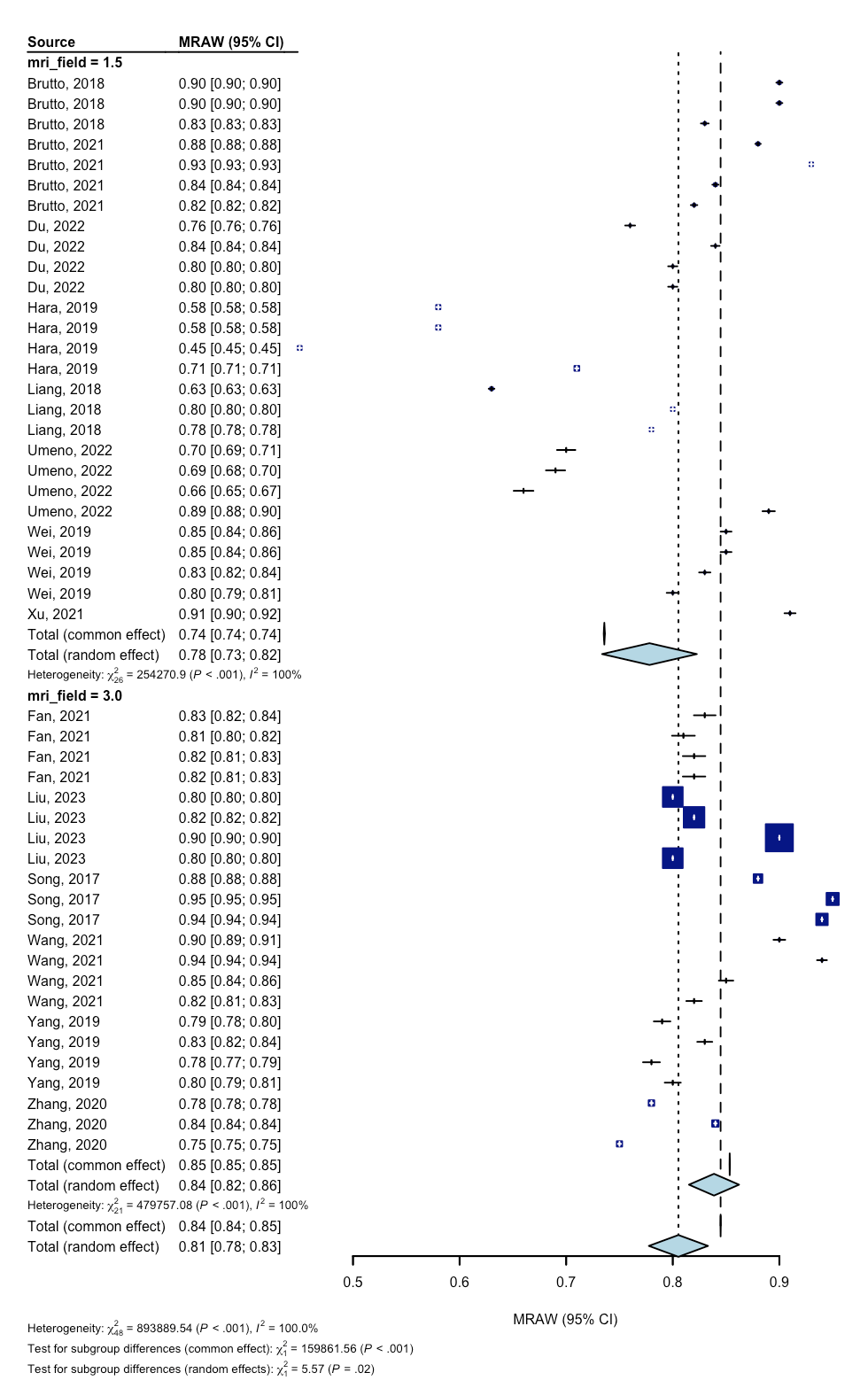


# **Figure S3.** Funnel plot for inter-rater reliability for each feature of the total cerebral small vessel disease score. A: Lacunes of presumed vascular origin B: White matter hyperintensities of presumed vascular origin. C: Cerebral microbleeds. D: Enlarged perivascular spaces.


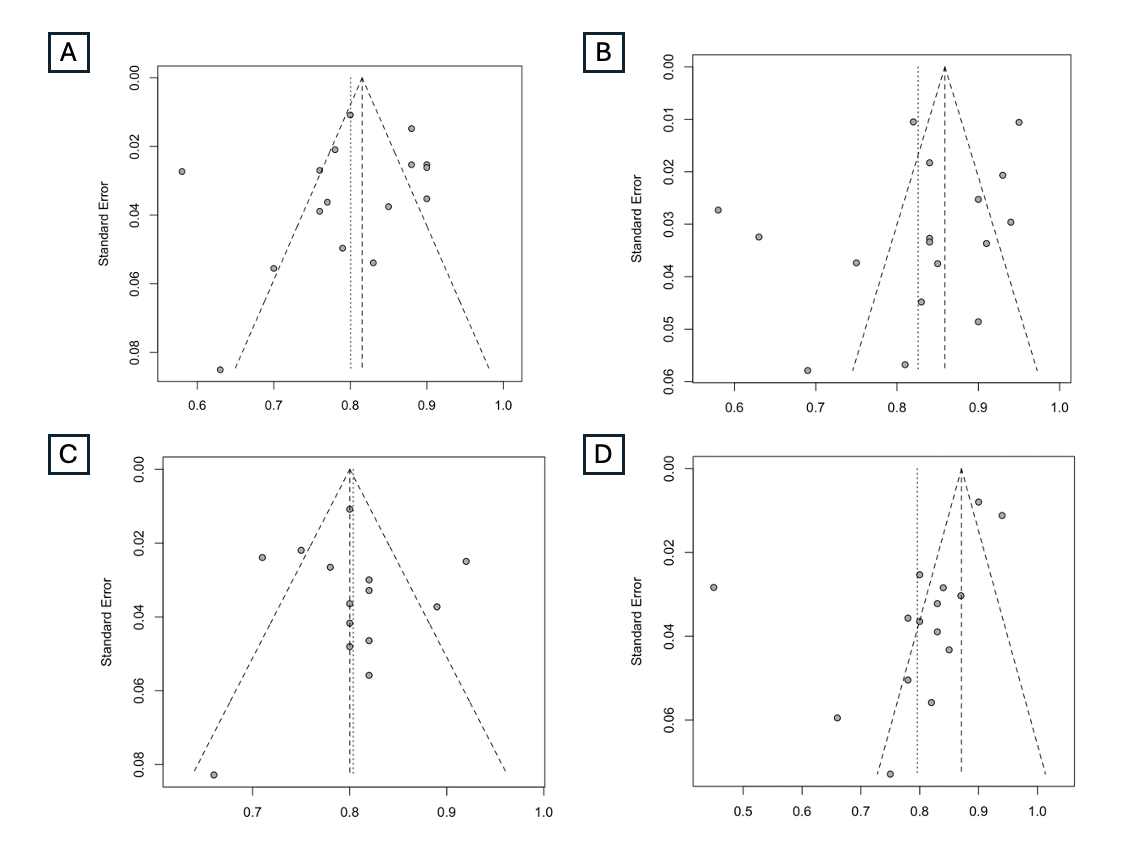


# **Figure S4.** Sensitivity analysis excluding studies about inter-rater reliability that contributed to significant asymmetry in the funnel plot (Hara, 2019 and Liang, 2018)


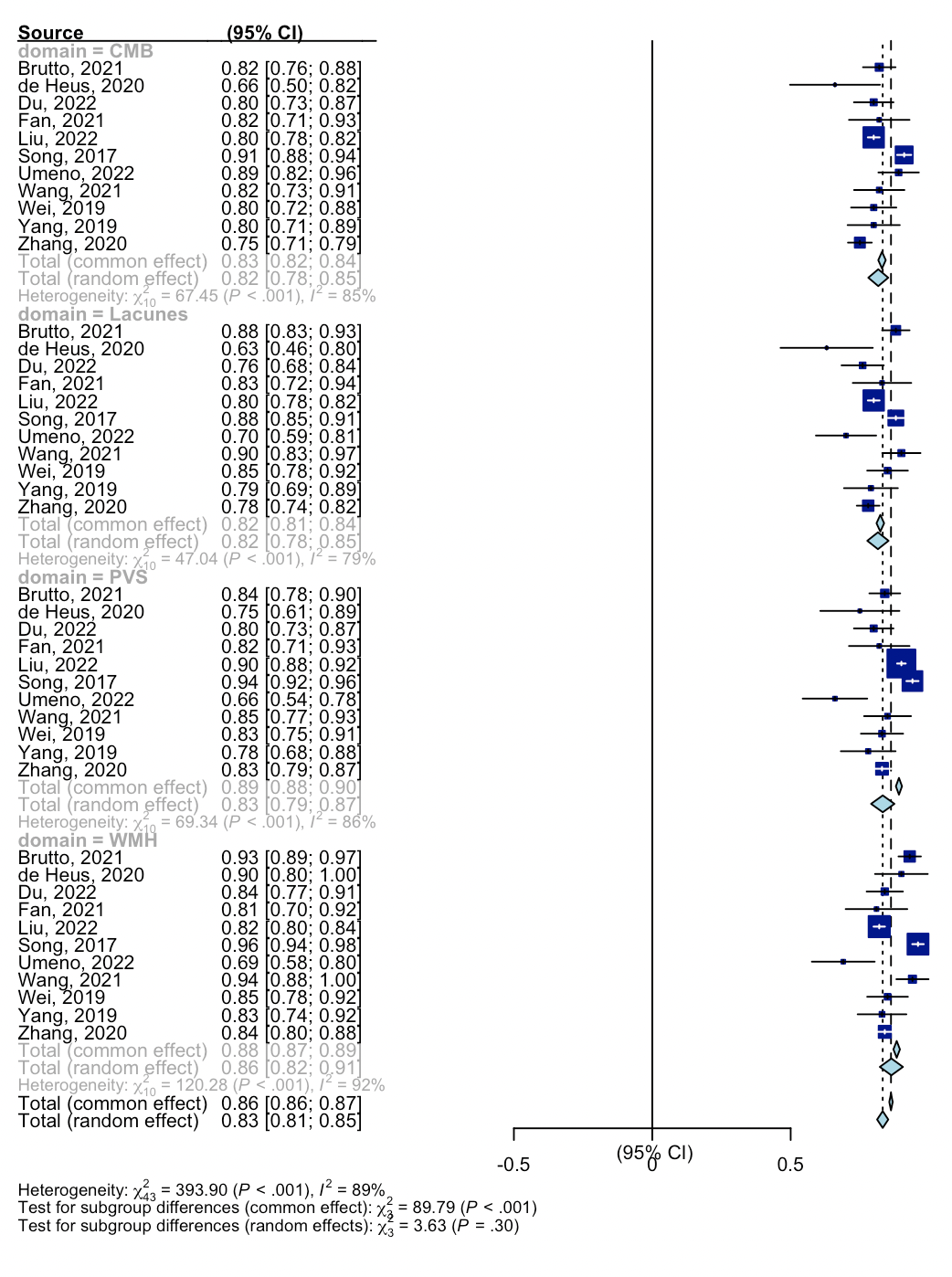


# **Figure S5.** Funnel plot of intra-rater reliability for each feature of the total cerebral small vessel disease score. A: Lacunes of presumed vascular origin B: White matter hyperintensities of presumed vascular origin. C: Cerebral microbleeds. D: Enlarged perivascular spaces.


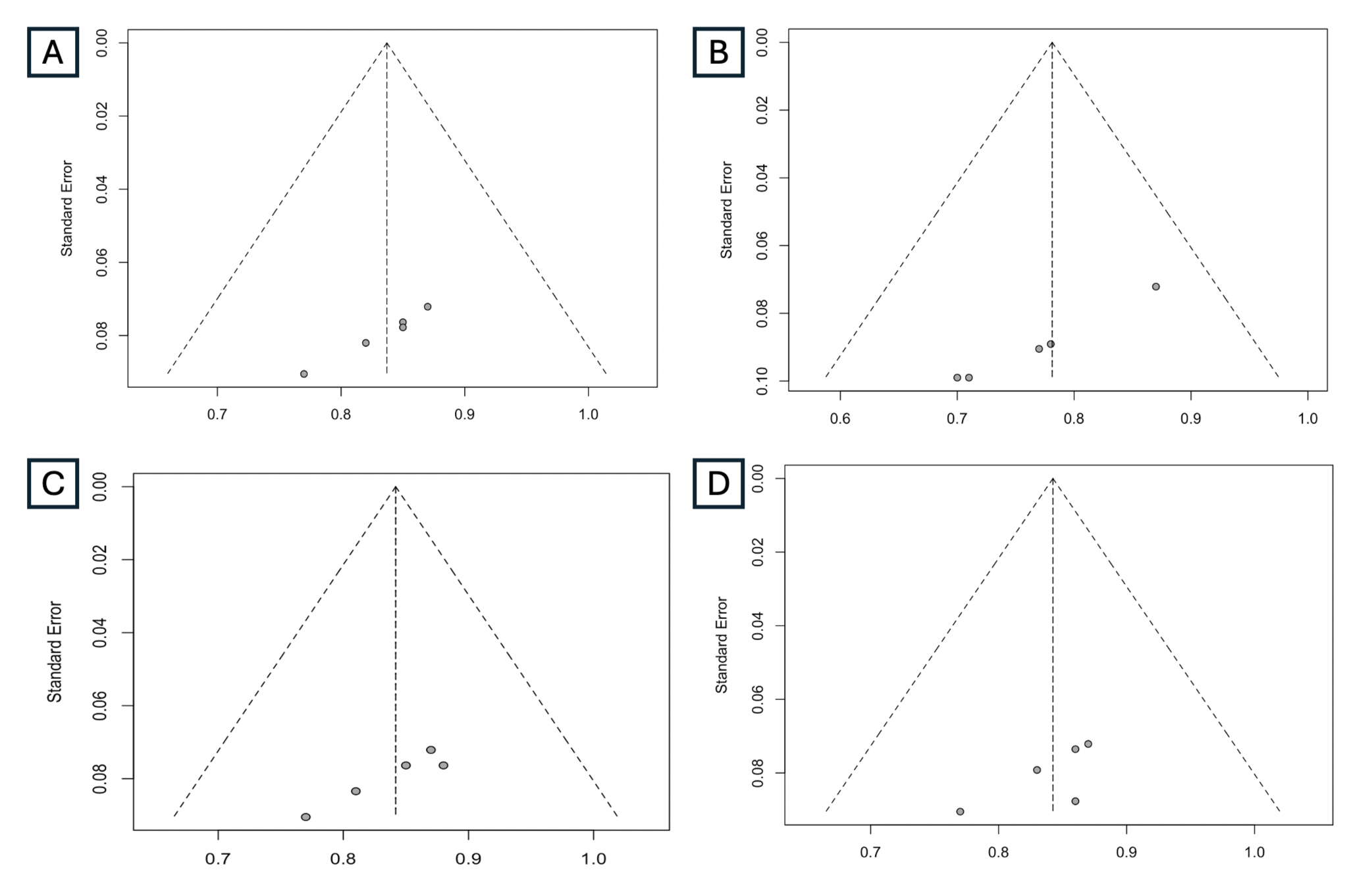


# **Table S1.** Studies reporting inter-rtater reliability of each domain of the total cerebral small vessel disease score

| **Study** | **Country (Mean or median age, % females)** | **Sample** | **Rater** | **Inter-rater reliability (Cohen’s kappa coefficients)** |
| --- | --- | --- | --- | --- |
| Brutto, 2021 | Ecuador (69 years, 56%) | 375 (Community-based study) | Two investigators  blinded to clinical data | CMB: 0.82 LAC: 0.88 PVS: 0.84 WMH: 0.93 |
| de Heus, 2020 | Netherlands (71 years, 34%) | 82 (Memory clinical population) | Two trained clinicians | CMB: 0.63 LAC: 0.75 PVS: 0.66 WMH: 0.9 |
| Du, 2022 | China (69 years, 33%) | 271 (Acute ischemic Stroke) | One clinician and one radiologist | CMB: 0.8 LAC: 0.76 PVS: 0.8 WMH: 0.84 |
| Fan, 2021 | China (72 years, 59%) | 289 (Outpatients with amnestic disorders) | Two neurologic radiologists who were blinded | CMB: 0.71 LAC: 0.88 PVS: 0.82 WMH: 0.81 |
| Hara, 2019 | Japan (59 years, 66%) | 858 (Healthy adults) | Researchers blinded to clinical data | CMB: 0.82 LAC: 0.88 PVS: 0.84 WMH: 0.93 |
| Liang, 2018 | China (67 years, 41%) | 563 (Acute ischemic stroke) | Two neurologists blinded to clinical data | CMB: 0.78 LAC: 0.76 PVS: 0.8 WMH: 0.63 |
| Liu, 2022 | China (61 years, 63%) | 3061 (Community-based study) | Two well-trained raters blinded to clinical data | CMB: 0.8 LAC: 0.8 PVS: 0.9 WMH: 0.82 |
| Song, 2017 | Korea (66 years, 38%) | 1096 (Acute ischemic stroke) | Two neurologists blinded to clinical information | CMB: 0.91 LAC: 0.88 PVS: 0.94 WMH: 0.96 |
| Umeno, 2022 | Japan (72 years, 38%) | 159 (Acute ischemic stroke and dialysis) | Neurosurgeon and neurologists blinded to clinical data | CMB: 0.89 LAC: 0.7 PVS: 0.66 WMH: 0.69 |
| Wang, 2021 | China (57 years, 41%) | 156 (Cognitively impaired patients) | Two neuroradiologists blinded to clinical data | CMB: 0.82 LAC: 0.9 PVS: 0.85 WMH: 0.94 |
| Wei, 2019 | China (68 years, 59%) | 207 (Acute ischemic stroke) | Two experienced researchers blinded to clinical  Data | CMB: 0.8 LAC: 0.85 PVS: 0.83 WMH: 0.85 |
| Yang, 2019 | China (68 years, 67%) | 156 (Healthy participants) | Two neurologists. Disagreements solved by neuroimaging expert | CMB: 0.8 LAC: 0.79 PVS: 0.78 WMH: 0.83 |
| Zhang, 2020 | China (60 years, 55%) | 904 (Community-based population) | Two neurologists blinded to clinical data | CMB: 0.75 LAC: 0.78 PVS: 0.83 WMH: 0.84 |

LAC: Lacunes of presumed vascular origin. WMH: White matter hyperintensities. CMB: Cortical microbleeds. PVS: Enlarged perivascular spaces.

# **Table S2**. Meta-regression for age, sex, and median score to identify predictors of heterogeneity of the reliability of the total small vessel disease score

| **Characteristic** | **R^2^** | **Age*** | **Sex*** | **Median tSVD score*** |
| --- | --- | --- | --- | --- |
| Lacunes | 0% | 0.59 | 0.98 | 0.79 |
| Microbleeds | 73% | **< 0.01**** | 0.93 | **<0.01**** |
| Enlarged perivascular spaces | 0% | 0.38 | 0.57 | 0.87 |
| White matter hyperintensities | 0% | 0.90 | 0.86 | 0.90 |

* P-value of the test of moderators. **: p < 0.05.

# **Table S3**. Studies reporting intra-rater reliability of each domain of the total cerebral small vessel disease score

| **Study** | **Country (Mean or median age, % females)** | **Sample size** | **Rater** | **Intra-rater reliability** |
| --- | --- | --- | --- | --- |
| Wei, 2019 | China (68 years, 59%) | 50 out of 207 (Random) | Single researcher’s (Neurology department) repeat assessment | At least 0.87 for each one of the four domains of the total cerebral small vessel disease score |
| Hara, 2019 | Japan (59 years, 66%) | 50 out of 858 (Random) | Single-researcher’s repeat assessment | Cohen’s kappa of the total cerebral small vessel disease was between  0.66 – 0.87 for each domain |
| Lau, 2018 | United Kingdom (69 years, 48%) | 50 out of 587  (Random) | One neuroradiologist provided ongoing supervision | LAC: 0.85 WMH: 0.78 CMB: 0.85 PVS: 0.83 |
| Lau, 2017 | United Kingdom and China (69 years, 44%) | Two sets of 50 out of 2002 (Random) | Two neuroradiologists provided ongoing supervision | LAC: 0.85 (UK), 0.82 (CH) WMH: 0.71 (UK), 0.70 (CH) CMB: 0.88 (UK), 0.81 (CH)  PVS: 0.86 (UK), 0.77 (CH) |

LAC: Lacunes of presumed vascular origin. WMH: White matter hyperintensities. CMB: Cortical microbleeds. PVS: Enlarged perivascular spaces. UK: United Kingdom and CH: China.

# **Table S4.** Characteristics of studies reporting associations between the total cerebral small vessel disease score, age and hypertension.

| **Study** | **Country (Mean or median age, % females)** | **Sample size** | **Mean age (years)** | **Sex (% Males)** | **Main results** |
| --- | --- | --- | --- | --- | --- |
| Dobrynina, 2022 | Russia (60 years, 70%) | 70 (53 CSVD +, 17 healthy patients) | 58 | 30 | Correlation between tSVD and mean awake systolic blood pressure: R^2^ = 0.23, p < 0.05 |
| Goldstein, 2019 | United States (62 years, 50%) | 449 (Cerebrovascular Diseases Registry) | 62 | 49.8 | Association between tSVD score and median age (p < 0.01): tSVD = 0: 55; tSVD = 1: 64; tSVD = 2: 72; tSVD = 3 or 4: 76 years  Association between tSVD score and hypertension (p < 0.01):  tSVD = 0: 51%; tSVD = 1: 68%, tSVD = 2: 81%; tSVD = 3 or 4: 87% |
| Hara, 2019 | Japan (59 years, 66%) | 858 (Neurologically healthy adults) | 59 | 43.8 | Association between tSVD score and age:  OR 1.13; 95% CI 1.09–1.17, p < 0.001  Association between tSVD score and hypertension:  OR 5.67; 95% CI 3.32–9.67, p < 0.05 |
| Heus, 2020 | Australia (71 years, 34%) | 82 (Memory clinic) | 71 | 66 | Association between tSVD score and median age (p < 0.01): tSVD = 0: 65, tSVD = 1: 69 , tSVD = 2: 74, tSVD = 3 or 4: 76 years Hypertension (%) per tSVD score value (p = 0.002):  tSVD = 0: 33%, tSVD = 1: 85%, tSVD = 2: 43%, tSVD = 3 or 4: 74% |
| Liu, 2022 | United Kingdom (61 years, 63%) | 3061 (Community-dwelling residents) | 61 | 46,5 | Association between tSVD score and Life’s simple 7 (LS7):  OR 0.73; 95% CI 1.13-2.93 |
| Staals,  2014 | United Kingdom (72 years, 48%) | 461 (Prospective stroke studies) | 68 | 62 | Association between tSVD score and age (per year):  OR 1.10, 95% CI 1.08-1.12 Association between tSVD score and hypertension:  OR 1.50, 95% CI 1.02-2.20 |
| Wiseman, 2016 | United Kingdom (51 years, 92%) | 51 (SLE patients) | 49 | 8 | Association between tSVD score and age:  OR 1.05; 95% CI 1.01–1.09  Association between tSVD score and hypertension: OR 1.82; 95% CI 1.13–2.93 |
| Yakushiji, 2018 | Japan (57 years, 54%) | 1451 (Neurologically healthy adults) | 57 | 46.5 | Association between tSVD score and age:  OR 1.78; 95% CI 1.55-2.05  Association between tSVD score and hypertension:  OR 2.47, 95% CI 1.93-3.15 |

LS7 is summary score is a quantitative measure of cardiovascular risk, that includes hypertension. CSVD: Cerebral Small Vessel Disease. tSVD: Total Cerebral Small Vessel Disease score.

# **Table S5.** Characteristics of studies reporting associations between total cerebral small vessel disease score, stroke, cognitive impairment, and cognitive tests

| **Study** | **Country (Median and mean age, % of females)** | **Sample size** | **Mean age (years)** | **Sex (% males)** | **Main results** |
| --- | --- | --- | --- | --- | --- |
| Brutto, 2017 | Ecuador (70 years, 58%) | 331 (Community-dwelling older adults) | 70 | 42 | Correlation between tSVD score and MoCA:  R^2^ = 0.328, p = 0.015 |
| Fan, 2021 | China (72 years, 59%) | 289 (Memory clinic) | 72 | 41 | Correlation between tSVD score and MoCA:  R = −0.377, p < 0.001 |
| Goldstein, 2019 | United States of America (62 years, 50%) | 449 (Mayo Clinic Florida Familial Cerebrovascular Diseases Registry) | 62 | 49.8 | Association of tSVD score with time to recurrent stroke (ischemic and hemorrhagic) and dementia:  tSVD = 1: HR 1.31 (CI 95% 0.75-2.26); tSVD = 2: HR .74 (CI 95% 0.95-3.2); tSVD = 3 or 4: HR 2.25 (CI 95% 1.10-4.61) |
| Guo, 2021 | China (67 years, 45%) | 275 (CSVD patients) | 68 | 55 | Vascular cognitive impairment is associated with tSVD score (OR 1.85, 95% IC 1.28–2.67, p < 0.001) |
| Han, 2021 | China (56 years, 63%) | 1082 (population-based study) | 56 | 37 | Association between tSVD score was associated with stroke:  tSVD score 2-4 versus 0: HR=12.73 (4.83–33.53) |
| Lau, 2017 | United Kingdom (69 years, 44%) | 2002 (Prospective register OXVASC \| HKU) | 69 | 55 | Association between tSVD score and stroke:  Ischemic stroke: OR 1.32, CI 95% 1.16–1.51, p < 0.0001 Intracerebral hemorrhage OR 1.54, CI 95% 1.11–2.13, p = 0.009 |
| Liu, 2021 | China (69 years, 46%) | 199 (CSVD patients) | 69 | 54 | Association between tSVD score and vascular cognitive impairment: OR: 1.414, 95% CI: 1.213–2.278, p < 0.05 |
| Pasi, 2021 | France (70 years, 47%) | 612 (ICH survivors) | 71 | 53 | Association between tSVD score and dementia risk:  HR 1.35, 95% CI 1.10-1.65, p < 0.05 |
| Shen, 2022 | China (64 years, 41%) | 81 (Parkinson`s disease) | 64 | 59 | Association between tSVD score and cognitive impairment:  OR 1.55, 95% CI 1.07 – 2.27, p = 0.02 |
| Staals,  2014 | United Kingdom (72 years, 48%) | 461 (Prospective stroke studies) | 68 | 62 | Association between tSVD score and lacunar stroke: OR 2.45, 95% CI 1.70-354, p < 0.001 |
| Suzuyama, 2020 | Japan (58 years, 53%) | 1349 (Kashima study) | 58 | 47 | Association between tSVD score and cerebro-cardiovascular events: HR per unit: 2.17; 95% CI 1.36–3.46; P = 0.001 |
| Umeno, 2022 | Japan (72 years, 48%) | 159 (Stroke patients undergoing hemodyalisis) | 72 | 62 | Association between tSVD score and recurrent stroke at five years: HR per unit: 1.72; 95% CI 1.34-2.21; p < 0.001. |
| Wang, 2021 | China (57 years, 41%) | 156 (CSVD patients) | 57 | 92 | Association between tSVD score and cognitive impairment:  OR 1.94; CI 95% 1.43-2.63; p < 0.001 |
| Xiang, 2018 | China (62 years, 20%) | 358 (Neurosyphilis and controls) | 62 | 80 | Association between tSVD score and cognitive impairment: OR 3.80; 95% CI 1.80–8.01; p <.001 |
| Xie, 2024 | China (65 years, 38%) | 126 (CSVD patients) | 66 | 62 | Association between tSVD score and motoric-cognitive syndrome:  tSVD = 1 compared with tSVD = 3: OR 0.228; 95% CI 0.07-0.70; p = 0.01. |
| Xu, 2021 | China (66 years, 66%) | 69 (CSVD patients) | 66 | 68 | Association between higher tSVD score and the presence of cognitive impairment: Median tSVD score 2 (1,3) x Median tSVD score 3 (2,4), p = 0.004 |

MoCA: Montreal Cognitive Assessment (MOCA). CSVD: Cerebral Small Vessel Disease. tSVD: Total cerebral small vessel disease. CAA: Cerebral Amyloid Angiopathy.
